# Longitudinal Effects of Coping Strategies on Mental Health of Older Adults Living Alone During the COVID-19 Pandemic

**DOI:** 10.1093/geroni/igab046.1704

**Published:** 2021-12-17

**Authors:** Seoyoun Kim, Hyunwoo Yoon, Yuri Jang

**Affiliations:** 1 Texas State University, San Marcos, Texas, United States; 2 Gongju National University, Gongju, Ch'ungch'ong-namdo, Republic of Korea; 3 University of Southern California, Los Angeles, California, United States

## Abstract

The COVID-19 pandemic and related social distancing measures have posed a significant threat to the mental health of older adults, particularly those living alone. Accordingly, the World Health Organization implemented the #HealthyAtHome program, encouraging people to keep in regular contact with loved ones, stay physically active, and keep a regular routine. The current study aims to examine a micro-longitudinal link between positive coping strategies (e.g., exercise, meditation, relaxation, and virtual social contacts) and depressive symptoms among older adults who live alone during the COVID-19 pandemic. We used 21 biweekly waves of longitudinal data from the Understanding America Study (UAS) collected between April 2020 and February 2021 (N=839, observation= 16,256). The multilevel models with correlated random effects were estimated to examine lagged effects of coping strategies (t-1) on depressive symptoms (t). The analysis used the xthybrid command with clustered standard errors in Stata 15.1. The results show that exercise (b=-.10, p=0.02), relaxation (b=-02, p=0.01), and virtual social contacts (b=-.01, p=0.01) were predictive of lower depressive symptoms even after controlling for time-invariant and time-varying covariates. Meditation, however, was associated with higher depressive symptoms (b=.01, p=0.02). The results show that modifiable lifestyle factors, such as taking time to exercise or relax, may enhance mental health and well-being for older adults living alone. Virtual social contacts such as video calls could be an effective way to keep older adults socially connected and emotionally healthy.

